# Beneficial Cardiac Structural and Functional Adaptations After Lumbosacral Spinal Cord Epidural Stimulation and Task-Specific Interventions: A Pilot Study

**DOI:** 10.3389/fnins.2020.554018

**Published:** 2020-10-22

**Authors:** Bonnie E. Legg Ditterline, Shelley Wade, Beatrice Ugiliweneza, Narayana Sarma Singam, Susan J. Harkema, Marcus F. Stoddard, Glenn A. Hirsch

**Affiliations:** ^1^Kentucky Spinal Cord Injury Research Center, University of Louisville, Louisville, KY, United States; ^2^Department of NeuroSurgery, University of Louisville, Louisville, KY, United States; ^3^Division of Cardiovascular Medicine, Department of Medicine, University of Louisville, Louisville, KY, United States; ^4^Division of Cardiology, Department of Medicine, National Jewish Health, Denver, CO, United States

**Keywords:** spinal cord injury, epidural stimulation, cardiac structure and function, systolic function, diastolic function, left ventricular structure

## Abstract

Cardiac myocyte atrophy and the resulting decreases to the left ventricular mass and dimensions are well documented in spinal cord injury. Therapeutic interventions that increase preload can increase the chamber size and improve the diastolic filling ratios; however, there are no data describing cardiac adaptation to chronic afterload increases. Research from our center has demonstrated that spinal cord epidural stimulation (scES) can normalize arterial blood pressure, so we decided to investigate the effects of scES on cardiac function using echocardiography. Four individuals with chronic, motor-complete cervical spinal cord injury were implanted with a stimulator over the lumbosacral enlargement. We assessed the cardiac structure and function at the following time points: (a) prior to implantation; (b) after scES targeted to increase systolic blood pressure; (c) after the addition of scES targeted to facilitate voluntary (i.e., with intent) movement of the trunk and lower extremities; and (d) after the addition of scES targeted to facilitate independent, overground standing. We found significant improvements to the cardiac structure (left ventricular mass = 10 ± 2 g, *p* < 0.001; internal dimension during diastole = 0.1 ± 0.04 cm, *p* < 0.05; internal dimension during systole = 0.06 ± 0.03 cm, *p* < 0.05; interventricular septum dimension = 0.04 ± 0.02 cm, *p* < 0.05), systolic function (ejection fraction = 1 ± 0.4%, *p* < 0.05; velocity time integral = 2 ± 0.4 cm, *p* < 0.001; stroke volume = 4.4 ± 1.5 ml, *p* < 0.01), and diastolic function (mitral valve deceleration time = -32 ± 11 ms, *p* < 0.05; mitral valve deceleration slope = 50 ± 25 cm s^–1^, *p* < 0.05; isovolumic relaxation time = −6 ± 1.9 ms, *p* < 0.05) with each subsequent scES intervention. Despite the pilot nature of this study, statistically significant improvements to the cardiac structure, systolic function, and diastolic function demonstrate that scES combined with task-specific interventions led to beneficial cardiac remodeling, which can reverse atrophic changes that result from spinal cord injury. Long-term improvements to cardiac function have implications for increased quality of life and improved cardiovascular health in individuals with spinal cord injury, decreasing the risk of cardiovascular morbidity and mortality.

## Introduction

Cardiac myocyte atrophy and the resulting decreases to the left ventricular mass and dimensions are well documented in spinal cord injury (Kessler et al., 1986; Eysmann et al., 1995; Gondim et al., 2004; Claydon et al., 2006; de Groot et al., 2006; Matos-Souza et al., 2011; Hostettler et al., 2012; Driussi et al., 2014; Williams et al., 2019). There is evidence that these reduced structural outcomes result from persistent decreases to preload and afterload: chronic skeletal muscle unloading can rapidly decrease the left ventricular volumes, mass, and contraction velocity in able-bodied and individuals with spinal cord injury (Arbeille et al., 2001; Meck et al., 2001; Martin et al., 2002; Summers et al., 2005; Giangregorio and McCartney, 2006; Platts et al., 2009; Moore et al., 2018). Decreased functional outcomes can be caused by sympathetic impairment, such that cardiac response during stress or exercise is significantly diminished in individuals with spinal cord injury when compared with able-bodied individuals (Teasell et al., 2000; Furlan et al., 2003; Grigorean et al., 2009; Theisen, 2012; Bartholdy et al., 2014; Wecht and Bauman, 2018). Therapeutic interventions that increase preload can increase the chamber size and improve the diastolic filling ratios in individuals with spinal cord injury, while athletes with spinal cord injury demonstrate greater left ventricle dimensions and improved relaxation velocities. This suggests that structural decreases in spinal cord injury are dynamic and can adapt to exercise interventions similar to non-injured individuals (Nash et al., 1991; Turiel et al., 2011; Maggioni et al., 2012; Guilherme et al., 2014). However, there are no data describing cardiac adaptation to interventions that lead to chronic afterload increases in individuals with spinal cord injury. Vascular stiffening and systemic inflammation are common in spinal cord injury, all of which can ultimately increase afterload and are implicated in diastolic dysfunction in able-bodied individuals (Bauman and Spungen, 2007, 2008; Gibson et al., 2008; West et al., 2013). Investigation of cardiac adaptation to increased afterload is therefore necessary, particularly as new research from our center has demonstrated that spinal cord epidural stimulation (scES) can normalize arterial blood pressure and mitigate orthostatic hypotension (Aslan et al., 2018; Harkema et al., 2018a; Legg Ditterline et al., 2020). Restoration of cardiovascular function at rest and during orthostatic stress would dramatically increase cardiac demand as individuals with spinal cord injury live with cardiac adaptation to persistent hypotension (Ditterline et al., 2020). Thus, we decided to investigate the effects of scES on cardiac structure and function. We hypothesized, first, that scES targeted to alleviate hypotension would lead to alterations in cardiac structure and function due to greater afterload from increased arterial blood pressure, reported previously (Aslan et al., 2018; Harkema et al., 2018a,b), and, second, that the addition of non-weight-bearing and weight-bearing motor interventions would alter the cardiac structure and function outcomes due to increased preload from the activation of the lower extremity and trunk muscles, with weight-bearing interventions eliciting the greatest improvements.

## Materials and Methods

### Participants

Included in this study were four individuals (three males and one female) with chronic, cervical motor-complete spinal cord injury (Table 1). Individuals were clinically stable, presented with orthostatic hypotension, persistent low resting blood pressure, and periodic symptoms of autonomic dysreflexia without evidence of cardiovascular disease unrelated to spinal cord injury. This research study was approved by the University of Louisville Institutional Review Board in accordance with the Declaration of Helsinki. Individuals provided written informed consent in order to participate (NCT-02037620).

**TABLE 1:** Demographic characteristics of the individuals at implant.

**ID**	**Age range (years)**	**Time since injury (years)**	**Level**	**AIS**
A41	21–25	7.2	C4	A
A68	31–35	3.8	C5	A
A80	31–35	7.9	C6	A
B21	31–35	6.9	C4	B

*AIS, American Spinal Injury Association impairment scale.*

### Echocardiography

Individuals lay in the left lateral decubitus position and were given sufficient time to acclimate prior to recording. Brachial blood pressure was recorded from the right arm. Individuals did not consume caffeine, alcohol, nicotine, or blood pressure medication the morning of the exam; they did not use scES for at least 12 h prior to acquisition to limit the residual effects of stimulation on the cardiovascular system. Assessments were repeated twice, with 2–4 days in between, to account for changes in volume related to variability in blood pressure. Registered diagnostic cardiac sonographers recorded images on a Philips EPIQ 7 ultrasound system with a Philips X5-1 MHz xMATRIX array transducer or a GE LOGIQ P6 ultrasound system with a GE 3Sp-D phased array transducer. Images were obtained in the parasternal long axis, parasternal short axis, and apical two-, three-, four-, and five-chamber views according to the standards and recommendations of the American Society of Echocardiography (Lang et al., 2015; Nagueh et al., 2016). Aortic, left ventricular, and left atrial dimensions were obtained using two-dimensional guided M-mode echocardiography. Left ventricular outflow velocities were measured using pulsed-wave Doppler recorded from the left ventricular outflow tract. Mitral inflow velocities during early (E-wave) and late (A-wave) diastole were recorded from the mitral valve leaflet tips using pulsed-wave Doppler. The isovolumic relaxation time was measured as the time between aortic valve closure and mitral valve opening. The myocardial peak systolic (*s*′) and early diastolic (*e*′) contraction velocities were measured using tissue Doppler imaging (TDI) in the lateral and septal annulus. Four consecutive cardiac cycles were recorded for off-line analysis.

Images were accepted for analysis according to the standards and recommendations of the American Society of Echocardiography (Lang et al., 2015; Nagueh et al., 2016). End-systolic volume, end-diastolic volume, and ejection fraction were calculated using Simpson’s biplane method of discs from the apical two- and four-chamber views. Cardiac output and stroke volume were calculated from the left ventricular outflow tract diameter and the velocity time integral (VTI) of blood flow measured from the parasternal long axis and five-chamber views, respectively. Left ventricular mass was estimated from the internal diastolic diameter, posterior wall dimension, and septal dimension (Schiller et al., 1989). Relative wall thickness of the left ventricle is calculated as the ratio of twice the posterior wall dimension to the internal diastolic diameter. Left atrial filling pressure was calculated as (1.24^∗^*E*/*e*′ ratio) +1.9 (Nagueh et al., 1997). Global longitudinal strain was calculated from the apical two-, three-, and four-chamber views. Global circumferential strain was measured from the parasternal short-axis view at basal, mid-, and apical depths.

### Implantation and Interventions

**FIGURE 1:**
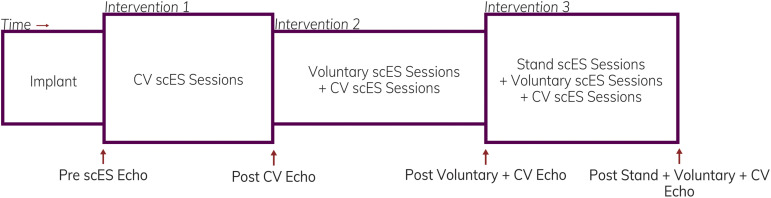
Representation illustrating the timeline of assessments and interventions. Echocardiography assessments were obtained in four individuals prior to starting spinal cord epidural stimulation (scES) and after completion of each intervention.

A 16-electrode array (5-6-5 Specify, Medtronic) was implanted under the T11-L1 vertebrae, spanning spinal cord segments L1–S1, as previously described (Harkema et al., 2011). Stimulation parameters, including electrode polarity, voltage, frequency, and pulse width, were unique to each individual and each intervention (below). We assessed the effects of scES interventions on cardiac function at the following time points (Figure 1): (a) prior to implantation; (b) after scES targeted to normalize systolic blood pressure (CV scES) (Harkema et al., 2018a,b); (c) after the addition of scES targeted to facilitate voluntary (i.e., with intent) movement of the trunk and lower extremities (Voluntary scES) (Angeli et al., 2014); and (d) after the addition of scES targeted to facilitate independent, overground standing (Stand scES) (Rejc et al., 2015). To prevent the reversal of functional gains between time points, interventions were added sequentially as individuals progressed through the study (Table 2). During the CV scES intervention, individuals trained only with CV scES. During the Voluntary scES intervention, individuals added in Voluntary scES and CV scES training sessions for a total of 4 h of stimulation each day. During the Stand scES intervention, individuals added Stand scES to Voluntary scES and CV scES training sessions, for a total of 5 h of stimulation each day. Individuals were given 1–4 h to rest in between scES sessions to minimize fatigue.

**TABLE 2:** Number of sessions that occurred during each intervention.

	**Intervention 1**	**Intervention 2**		**Intervention 3**		
	**CV scES**	**Voluntary scES**	**+ CV scES**	**Stand scES**	**+ Voluntary scES**	**+ CV scES**
Sessions (mean ± SD)	89 ± 13	95 ± 15	96 ± 52	80 ± 7	120 ± 31	102 ± 35

*Data reported are the mean ± SD from four individuals. *scES*, spinal cord epidural stimulation; *CV scES*, scES targeted to normalize systolic blood pressure; *Voluntary scES*, scES targeted to facilitate voluntary movement of the trunk and lower extremities; *Stand scES*, scES targeted to facilitate independent, overground standing.*

The stimulation parameters for CV scES were identified specifically to increase systolic blood pressure within a normative range (105–120 mmHg) without activation of the skeletal muscle, demonstrated by the absence of EMG activity (Harkema et al., 2018a,b). Individuals utilized CV scES in the sitting position for 2 h each day, during which time systolic blood pressure was maintained within the targeted range. Systolic blood pressure, diastolic blood pressure, and heart rate were monitored during each session to evaluate the effectiveness of the stimulation configuration and individual safety.

Stimulation configurations for the Voluntary scES interventions were selected to facilitate bilateral initiation, termination, and controlled movement of the trunk and lower extremities, including trunk flexion, extension, and rotation; isolated extension and flexion of the hip, knee, and ankle joints; and coordinated extension and/or flexion of the hip, knee, and ankle to move the lower extremities (Angeli et al., 2014). Individuals completed 2 h of daily Voluntary scES training while sitting or supine, alternating daily between trunk and lower extremity training sessions.

Stimulation configurations for Stand scES were selected to facilitate overground, independent, weight-bearing standing in a custom frame (Rejc et al., 2015). While the individual was seated, the stimulation amplitude was low to enable proprioception to coordinate the transition from sitting to standing; upon standing, the amplitude increased to the voltage optimized for each individual to stand fully weight-bearing. Manual assistance was provided at the hips or knees only if the joints moved outside the standing posture. Individuals were encouraged to stand as long as possible during the training sessions, up to a goal of 60 min. Individuals completed Stand scES each weekday.

### Statistics

Data were analyzed with mixed linear models in which each measurement was the outcome. The only independent variable was the experimental time point (Pre CV scES, Post CV scES, Post Voluntary scES + CV scES, and Post Stand scES + Voluntary scES + CV scES). For each participant, a random intercept and random slope for time point were included. Assessments were repeated at each time point, but measurements were only included for analysis if the images met the standards established by the American Society of Echocardiography; to account for such variability, we included this in the linear model as a random effect nested within time point. To evaluate the improvements over time, linear contrasts were built to compare Post scES time points with Pre CV scES and evaluated with a *t* test. The significance level was set to 0.05 and all tests were two-sided. Statistical analyses were performed in SAS 9.4 (SAS Inc, Cary, NC, United States).

## Results

**FIGURE 2:**
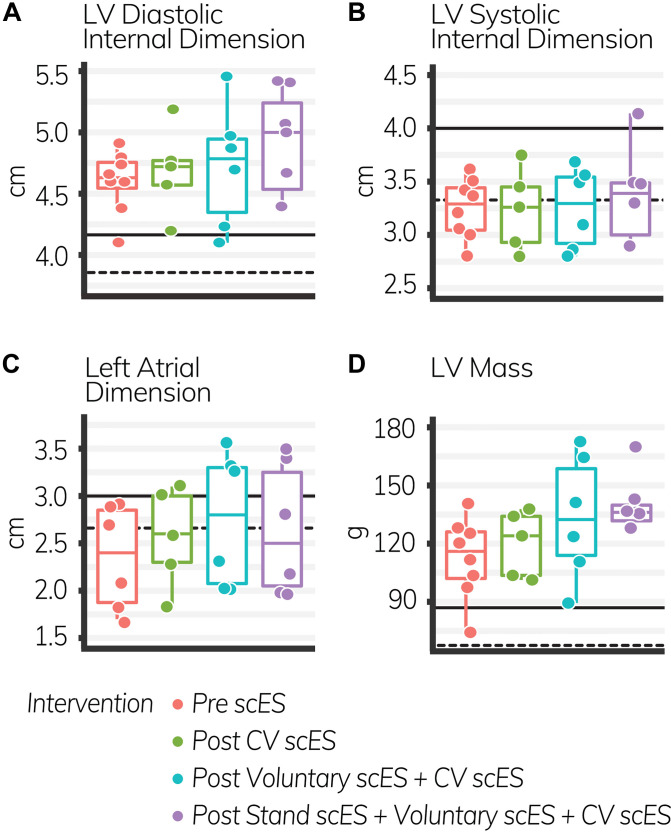
The LV diastolic internal dimension **(A)**, left atrial dimension **(C)**, and LV mass **(D)** increased significantly with each subsequent intervention (*p* < 0.05); LV systolic internal dimension **(B)** did not change. *Black lines* indicate the healthy limit according to the American Society of Echocardiography guidelines. LV diastolic internal dimension: females (*dashed line*), 3.8 cm; males (*solid line*), 4.2 cm; LV systolic internal dimension: females (*dashed line*), 3.3 cm; males (*solid line*), 4.0 cm; left atrial dimension: females (*dashed line*), 2.8 cm; males (*solid line*), 3.0 cm; LV mass: females (*dashed line*), 67 g; males (*solid line*), 88 g. Each *circle* is an individual observation. *Box-and-whisker plots* illustrate the 5th, 25th, 50th, 75th, and 95th percentiles. Each individual (*n* = 4) had two echocardiography assessments at each time point, but data were only included for analysis if they met the image standards set by the American Society of Echocardiography.

The mean age of individuals (*n* = 4) was 30.8 ± 2.7 years. At implant, the duration of injury was 6.5 ± 1.1 years. We found significant increases to the aortic root, left atrial dimension, and left ventricular chamber dimension and mass after scES interventions, associated with statistically significant increases in systolic and diastolic function measurements. After the Voluntary scES intervention, the left ventricular mass (Δ22 ± 7 g, *p* < 0.05), interventricular septum dimension (Δ0.16 ± 0.05 cm, *p* < 0.05), aortic root diameter (Δ0.11 ± 0.03 cm, *p* < 0.01), and left atrial dimension (Δ0.37 ± 0.14 cm, *p* < 0.05) increased significantly compared with the Pre scES time point. Likewise, after the Stand scES intervention, the left ventricular mass (Δ29 ± 7 g, *p* < 0.01) and the aortic root diameter (Δ0.12 ± 0.03 cm, *p* < 0.01) increased significantly compared with the Pre scES time point (Table 3). With each subsequent scES intervention, the left ventricular mass (10 ± 2 g, *p* < 0.001), left ventricular internal dimension during diastole (0.1 ± 0.04 cm, *p* < 0.05), left ventricular internal dimension during systole (0.06 ± 0.03 cm, *p* < 0.05), and the interventricular septum dimension (0.04 ± 0.02 cm, *p* < 0.05) increased significantly (Figure 2). The aortic root diameter (0.04 ± 0.01 cm, *p* < 0.001) and left atrial dimension (0.10 ± 0.04 cm, *p* < 0.05) also increased significantly with each subsequent scES intervention.

**TABLE 3:** Left side chamber size, geometry, and mass before and after spinal cord epidural stimulation (scES) and task-specific interventions.

	**Time points**				**Model results**	
	**Pre scES**	**Post CV scES**	**Post Voluntary scES + CV scES**	**Post Stand scES + Voluntary scES + CV scES**	**Estimate (SE)**	***p*-value**
Left ventricle mass (g)	113 (10)	124 (11)	135 (11)*	142 (10)*	10 (2)	**<0.001**
Left ventricle internal diameter during diastole (cm)	4.71 (0.2)	4.84 (0.2)	4.86(0.2)	5.02(0.2)	0.1(0.04)	**0.011**
Left ventricle internal diameter during systole (cm)	3.33 (0.19)	3.32 (0.19)	3.33(0.19)	3.52(0.19)	0.06(0.03)	**0.040**
Interventricular septum dimension (cm)	0.57 (0.07)	0.63 (0.07)	0.73(0.07)*	0.67(0.07)	0.04(0.02)	**0.021**
Left ventricle posterior wall dimension during diastole (cm)	0.86 (0.05)	0.89 (0.05)	0.88(0.05)	0.93(0.05)	0.02(0.01)	0.177
Relative wall thickness	0.38 (0.02)	0.37 (0.03)	0.37(0.02)	0.38(0.03)	0 (0.01)	0.784
Aortic root diameter (cm)	2.87 (0.08)	2.88 (0.08)	2.98(0.08)*	2.99(0.08)*	0.04(0.01)	**<0.001**
LVOT diameter (cm)	2.15 (0.14)	2.11 (0.14)	2.16(0.14)	2.12(0.14)	−0.01(0.01)	0.585
Left atrial dimension (cm)	2.37 (0.31)	2.68 (0.32)	2.74(0.31)*	2.64(0.31)	0.1(0.04)	**0.046**

Data are the estimate (SE) of the echocardiography data obtained from individuals with spinal cord injury (*n* = 4) before and after spinal cord epidural stimulation (scES) and task-specific interventions. Measurements from each intervention are compared to pre scES measurements (*p < 0.05). Changes associated with the addition of each subsequent scES intervention are presented as the estimated change (SE) and p value; significant changes are bolded. *LVOT*, left ventricular outflow tract; *CV scES*, scES targeted to normalize systolic blood pressure; *Voluntary scES*, scES targeted to facilitate voluntary movement of the trunk and lower extremities; *Stand scES*, scES targeted to facilitate independent, overground standing. Bolded values in the table correspond to *p*-values <0.05.

**FIGURE 3:**
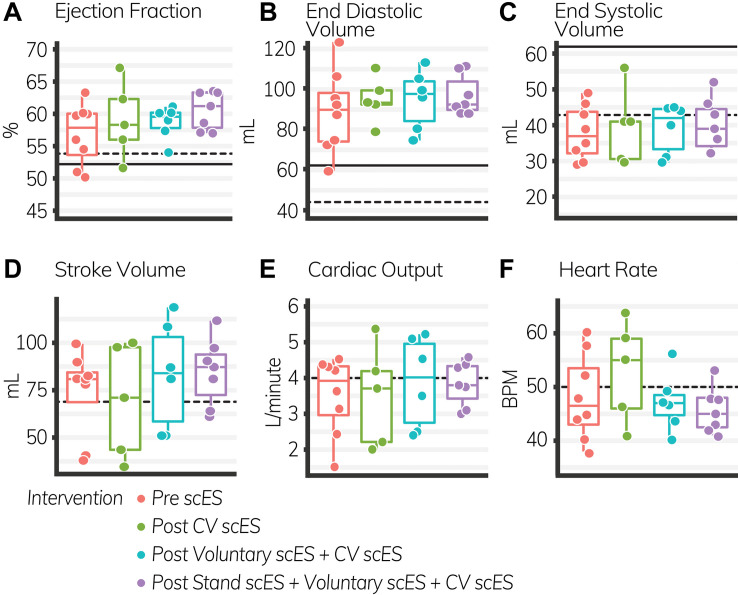
Global systolic function outcomes before and after spinal cord epidural stimulation (scES) interventions. With each subsequent scES intervention, the ejection fraction **(A)** and stroke volume **(D)** increased significantly (*p* < 0.05). End diastolic volume **(B)**, end systolic volume **(C)**, cardiac output **(E)**, and heart rate **(F)** did not change. *Black lines* indicate the healthy limit according to the American Society of Echocardiography guidelines. Ejection fraction: females (*dashed line*), 54%; males (*solid line*), 52%; end-diastolic volume: females (*dashed line*), 46 ml; males (*solid line*), 62 ml; end-systolic volume: females (*dashed line*), 42 ml; males (*solid line*), 61 ml; stroke volume: females and males (*dashed line*), 70 ml; cardiac output: females and males (*dashed line*), 4.0 L/min; heart rate: females and males (*dashed line*), 50 bpm. Each *circle* is an individual observation. *Box-and-whisker plots* illustrate the 5th, 25th, 50th, 75th, and 95th percentiles. Each individual (*n* = 4) had two echocardiography assessments at each time point, but data were only included for analysis if they met the image standards set by the American Society of Echocardiography.

The VTI of blood (i.e., distance traveled with each heartbeat, 4.4 ± 1.6 cm, *p* < 0.05) was significantly increased after the Stand scES intervention compared with the Pre scES time point (Table 4). With each subsequent scES intervention, the ejection fraction (1 ± 0.4%, *p* < 0.05) increased significantly without changes to the end-diastolic or end-systolic volume, but the VTI (2 ± 0.4 cm, *p* < 0.001) and stroke volume (4.4 ± 1.5 ml, *p* < 0.01) increased significantly without a change in heart rate (Figure 3). Diastolic blood pressure (4 ± 1.7 mmHg, *p* < 0.05) increased significantly with each subsequent scES intervention.

**TABLE 4:** Global systolic function and blood pressure outcomes before and after spinal cord epidural stimulation (scES) interventions.

	**Timepoint**				**Model Results**	
	**Pre scES**	**Post CV scES**	**Post Voluntary scES + CV scES**	**Post Stand scES + Voluntary scES + CV scES**	**Estimate (SE)**	**p-value**
Ejection fraction, %	57 (2)	58 (2)	58 (2)	60 (2)	1 (0.4)	**0.034**
End diastolic volume, mL	89 (7)	97 (7)	95 (8)	96 (7)	3 (2.0)	0.192
End systolic volume, mL	38 (4)	42 (4)	40 (4)	41 (4)	1 (0.7)	0.216
Velocity time integral, cm	20 (2)	21 (2)	23 (2)	25 (2)	2 (0.4)	**<0.001**
Cardiac output, L/min	3.8 (0.6)	4.1 (0.6)	4.2 (0.6)	4.2 (0.6)	0.2 (0.1)	0.051
Stroke volume, mL	73 (12)	77 (12)	84 (12)	84 (12)	4.4 (1.5)	**0.007**
Systolic blood pressure, mmHg	99 (10)	114 (12)	101 (11)	117 (11)	4 (3.1)	0.244
Diastolic blood pressure, mmHg	52 (5)	65 (6)	57 (6)	67 (6)	4 (1.7)	**0.034**
Heart rate, BPM	49 (3)	54 (3)	49 (4)	48 (4)	−1 (0.8)	0.378
s’ contraction velocity, cm/s	9 (0.9)	8 (0.8)	8 (0.8)	9 (0.8)	−0.2 (0.2)	0.409
Global circumferential strain, %	−21 (2)	−23 (2)	−23 (2)	−23 (2)	0.4 (0.4)	0.291
Global longitudinal strain, %	−25 (3)	−26 (3)	−26 (3)	−27 (3)	0.5 (0.3)	0.066

Data are the estimate (SE) of the echocardiography data obtained from individuals with spinal cord injury (*n* = 4) before and after scES and task-specific interventions. Measurements from each intervention are compared to pre scES measurements (*p < 0.05). Changes associated with the addition of each subsequent scES intervention are presented as the estimated change (SE) and *p* value. *CV*, cardiovascular; *CV scES*, scES targeted to normalize systolic blood pressure; *Voluntary scES*, scES targeted to facilitate voluntary movement of the trunk and lower extremities; *Stand scES*, scES targeted to facilitate independent, overground standing. Bolded values in the table correspond to *p*-values <0.05.

**FIGURE 4:**
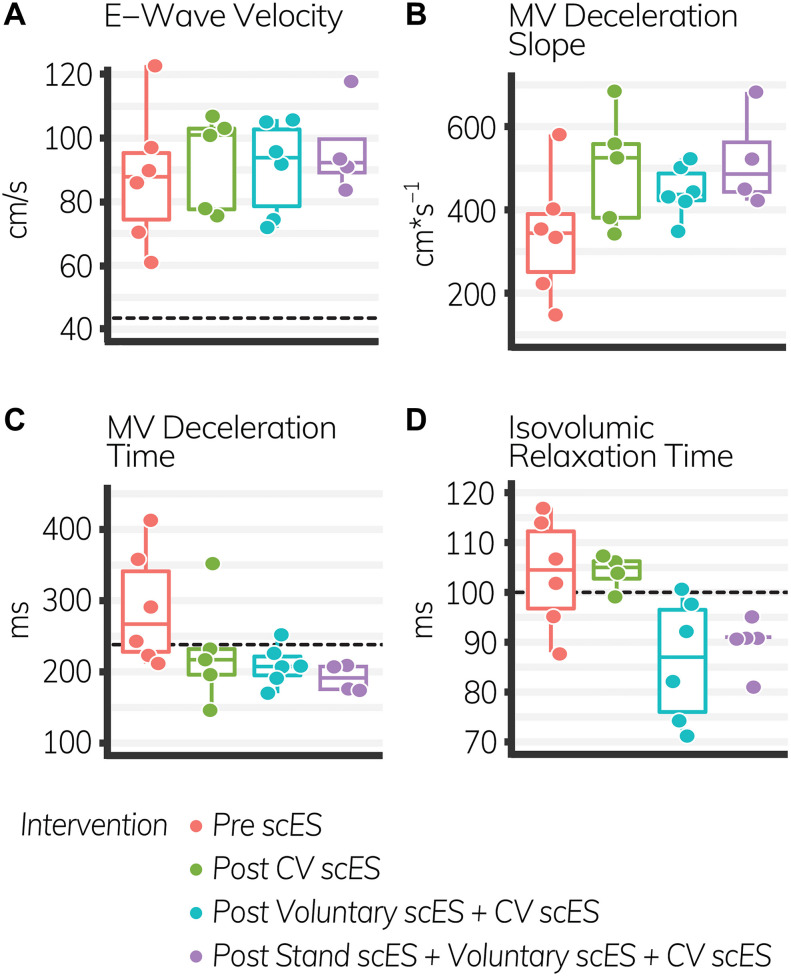
Diastolic function outcomes before and after spinal cord epidural stimulation (scES) interventions. With each subsequent intervention, the mitral valve (*MV*) deceleration slope **(B)**, increased significantly (*p* < 0.05), while the MV deceleration time **(C)**, and isovolumic relaxation time **(D)** decreased significantly (*p* < 0.05). E-wave velocity **(A)** did not change. *Black dashed lines* indicate the healthy limit according to the American Society of Echocardiography guidelines. *E*-wave velocity: females and males, 42 cm s^–1^; MV deceleration time: females and males, 140 ms; isovolumic relaxation time: females and males, 70 ms. The MV deceleration slope does not have a healthy range established by the American Society of Echocardiography. Each *circle* is an individual observation. *Box-and-whisker plots* illustrate the 5th, 25th, 50th, 75th, and 95th percentiles. Each individual (*n* = 4) had two echocardiography assessments at each time point, but data were only included for analysis if they met the image standards set by the American Society of Echocardiography.

The isovolumic relaxation time decreased significantly (Δ-18 ± 7 ms, *p* < 0.05) after the Voluntary scES intervention compared with the pre scES time point. The mitral valve deceleration slope increased significantly (Δ179 ± 78 cm s^–1^, *p* < 0.05) and the mitral valve deceleration time decreased significantly (Δ−98 ± 39 ms, *p* < 0.05) after the Stand scES intervention compared with the Pre scES time point (Table 5). With each subsequent scES intervention, the mitral valve deceleration time (−32 ± 11 ms, *p* < 0.05) and the isovolumic relaxation time (−6 ± 1.9 ms, *p* < 0.05) decreased significantly and the mitral valve deceleration slope (50 ± 25 cm s^–1^, *p* < 0.05) increased significantly (Figure 4). These changes were not associated with the changes to the *E*/*A* ratio, *e*′ velocity, *E*/*e*′ ratio, or left atrial filling pressure.

**TABLE 5:** Diastolic function outcomes before and after spinal cord epidural stimulation (scES) interventions.

	**Timepoint**				**Model Results**	

	**Pre scES**	**Post CV scES**	**Post Voluntary scES + CV scES**	**Post Stand scES + Voluntary scES + CV scES**	**Estimate (SE)**	**p-value**
Mitral valve peak E-wave velocity, cm/s	80 (11)	91 (9)	89 (10)	93 (10)	3 (2.6)	0.229
Mitral valve peak A-wave velocity, cm/s	51 (4)	52 (5)	54 (5)	50 (5)	0 (1.3)	0.999
E/A ratio	1.6 (0.4)	1.8 (0.3)	1.6 (0.4)	1.9 (0.3)	0.08 (0.1)	0.370
Mitral valve deceleration time, ms	290 (24)	229 (27)	209 (24)	192 (30)	−32 (11)	**0.012**
Mitral valve deceleration slope, cm*s^–1^	340 (50)	498 (54)	445 (50)	519 (60)	50 (25)	**0.048**
Isovolumic relaxation time, ms	104 (5)	104 (6)	85 (5)	89 (5)	−6 (1.9)	**0.008**
e’ relaxation velocity, cm/s	13 (1)	13 (1)	12 (1)	13 (1)	−0.1 (0.3)	0.728
E/e’ ratio	7.0 (1)	6.7 (1)	7.5 (1)	7.5 (1)	0.4 (0.3)	0.213
Left atrial filling pressure, mmHg	11 (1)	11 (1)	11 (1)	11 (1)	0.4 (0.3)	0.239

Data are the estimate (SE) of the echocardiography data obtained from individuals with spinal cord injury (*n* = 4) before and after scES and task-specific interventions. Measurements from each intervention are compared to pre scES measurements (*p < 0.05). Changes associated with the addition of each subsequent scES intervention are presented as the estimated change (SE) and *p* value. *CV*, cardiovascular; *CV scES*, scES targeted to normalize systolic blood pressure; *Voluntary scES*, scES targeted to facilitate voluntary movement of the trunk and lower extremities; *Stand scES*, scES targeted to facilitate independent, overground standing. Bolded values in the table correspond to *p*-values <0.05.

## Discussion

We found that significantly increased systolic function and diastolic function measures increased the left atrial and ventricular chamber and aortic root dimensions after scES interventions. With each subsequent scES intervention, the ejection fraction, stroke volume, and mitral valve deceleration slope increased significantly, while the isovolumic relaxation time and mitral valve deceleration time decreased significantly, suggestive of an improved systolic and diastolic function. The left ventricular mass, diastolic internal dimension, and systolic internal dimension also increased significantly. These statistically significant structural improvements suggest that scES interventions could lead to cardiac remodeling and reverse atrophic changes that result from spinal cord injury – the left ventricle dimensions and mass increased significantly, and all volume measurements were obtained without scES. The myocardial, systolic function, and diastolic function changes that occurred in four individuals with spinal cord injury (SCI) were thus adaptations to the scES interventions and not just residual effects of stimulation. Long-term improvements to cardiac function have implications for increased quality of life and improved cardiovascular health in individuals with spinal cord injury, decreasing the risk of cardiovascular morbidity and mortality.

### Structural and Functional Myocardial Improvements

Measurement of the left ventricle dimensions illustrates preload and afterload within the left ventricle and its ability to generate sufficient force to open the aortic valves against the high-resistance systemic circulation. After scES interventions, the left ventricle dimensions and mass increased significantly in response to increased preload and afterload. Even though these increases were not accompanied by significant increases to the posterior wall thickness, the relative wall thickness did not change after scES interventions. There was therefore no evidence of eccentric (i.e., maladaptive) hypertrophy of the myocardium because the geometry of the left ventricle remained the same after each intervention. In four individuals, the myocardium was thus able to strengthen appropriately in response to increased cardiac demand and wall stress imposed by the scES interventions. These changes are similar to those reported by other groups that observed improved posterior wall (Δ1.5 mm), interventricular septum (Δ1.6 mm), and left ventricular diastolic (Δ3.2 mm) dimensions in individuals with SCI after functional electrical stimulation. These changes also mirror those observed in non-injured individuals after reversal of left ventricular atrophy (mass = Δ12.4 g) and left ventricular hypertrophy (posterior wall = Δ1.7 mm, interventricular septum = Δ1.2 mm) (Nash et al., 1991; Gaddam et al., 2010; Westby et al., 2016). This beneficial adaptation has implications for long-term cardiovascular health in individuals with spinal cord injury, especially in light of new interventions that lead to long-term increases in preload and afterload. Maladaptive thinning of the myocardium can ultimately lead to systolic and diastolic dysfunction whereby the weakened left ventricle cannot adequately pump blood to maintain homeostasis (Cwajg et al., 2000; Thygesen et al., 2012; Hoit, 2014). It is therefore possible that scES interventions not only lead to the recovery of cardiovascular and motor function but could reverse myocardial atrophy and improve cardiac health (Harkema et al., 2011, 2018a,b; Angeli et al., 2014, 2018; Rejc et al., 2017a,b; Aslan et al., 2018).

Systolic function outcomes illustrate the strength of the left ventricle as it pumps blood into the systemic circulation. We found significant increases to the ejection fraction and stroke volume with each subsequent scES intervention, suggesting a long-term adaptation to increased cardiac demand in four individuals with SCI. This observation is similar to other groups that report systolic function increases after body weight-supported treadmill training (Turiel et al., 2011). These functional increases persisted without active stimulation, and, unlike previous studies, we found significantly increased systolic function outcomes despite significant increases to arterial blood pressure. These significant increases were independent of load or heartbeat because they were not associated with increases in preload or filling time. Increased strength of the left ventricle is also illustrated by stable end-systolic volume with each scES intervention because, with each heartbeat, a greater amount of blood is pumped into the systemic circulation despite a dramatically increased afterload (Aslan et al., 2018; Harkema et al., 2018a,b). Each heartbeat thus removes a greater amount of blood from the compliant venous circulation, increasing oxygen delivery to and waste removal from metabolically active tissues. This is particularly beneficial to the brain and heart as they possess the greatest metabolic demand and carry significant risk of adverse event when hypoperfused or oxygen-deprived (Yarkony et al., 1986; Bisharat et al., 2002; Dolinak and Balraj, 2007; Jegede et al., 2010; Wu et al., 2012; Wecht and Bauman, 2013; Phillips et al., 2014; Katz and Rolett, 2016; Wecht et al., 2018). Restoration of the ejection fraction and stroke volume in four individuals with spinal cord injury carries additional significance when considering the degree to which cardiovascular dysregulation decreases quality of life. Risk of syncope and persistent fatigue significantly delay therapeutic interventions, restrict independence and autonomy, and limit social engagement (Barrett-Connor and Palinkas, 1994; Blackmer, 1997; Illman et al., 2000; Furlan and Fehlings, 2008; Carlozzi et al., 2013; Guilcher et al., 2013; Piatt et al., 2016). Significant improvements to systolic function after scES interventions thus have the potential to improve the overall health, improve quality of life, and decrease the risk of cardiovascular disease in individuals with spinal cord injury.

Diastolic function outcomes quantify the elasticity of the ventricles and the degree to which the myocardium stretches during diastole to enable passive filling. With each subsequent scES intervention, we found significant increases to the left atrial dimension and mitral valve deceleration slope and associated decreases to the mitral valve deceleration time, illustrating greater preload and blood velocity through the mitral valve during early (i.e., passive) diastole (Stoddard et al., 1989). This was not associated with increased left atrial filling pressure or the *E*/*e*′ ratio, nor with decreases to *e*′ velocity. Therefore increased blood velocity through the mitral valve did not result from maladaptive increased left atrial pressure “pushing” blood into the left ventricle, but rather from stretch of the left ventricle “pulling” blood from the left atrium (Park and Marwick, 2011; Oliveira et al., 2014). Moreover, diastolic improvements in these four individuals persisted without active stimulation, suggesting an adaptation to scES interventions that led to long-term increases in venous return. Greater preload during early diastole provides a greater stroke volume without associated increases in pathologic metabolic demand, maintains left ventricular elasticity, and potentially decreases risk of heart failure (Redfield et al., 2003).

There were also significant decreases to the isovolumic relaxation time after scES interventions, similar to improvements observed after body weight-supported treadmill training, indicating improved coronary perfusion and cardiac health in these four individuals with SCI (Turiel et al., 2011). Prolonged isovolumic relaxation time precedes diastolic dysfunction and develops as increased afterload delays cross-bridge inactivation, preventing complete relaxation throughout the myocardium during diastole. Incomplete relaxation of the left ventricle during diastole, illustrated by increased isovolumic relaxation time and *E*/*e*′ ratio, prevents the decrease in pressure required to open the mitral valve (Gillebert and Lew, 1991; Leite-Moreira et al., 1999; Cheng et al., 2009; Parikh et al., 2016; Taqueti et al., 2018). This decreases coronary perfusion in areas where cross-bridges remain active. Myocardial contraction during systole compresses the microvasculature such that coronary blood flow velocity is greatest during diastole (Galderisi et al., 2008; Altunkas et al., 2014). Significant decreases to the isovolumic relaxation time after scES suggest more rapid cross-bridge inactivation and more rapid onset of complete relaxation, which could increase coronary perfusion. It is therefore significant that we found significant improvements to the isovolumic relaxation time in these four individuals with SCI – despite significant increases to afterload – because significant improvements to coronary perfusion have serious implications for cardiac health. Increasing oxygen delivery to the myocardium decreases the maladaptive signaling pathways that cause concentric or eccentric hypertrophy and ultimately lead to heart failure (Ingwall, 2009; Katz and Rolett, 2016). This indicates that scES interventions may lead to beneficial improvements in coronary perfusion and could decrease the risk of developing myocardial ischemia.

### Effects of Epidural Stimulation

While there was no investigation into the mechanism in this research study, animal models may lend insight into the hemodynamic changes that occur upon active stimulation. Research using anesthetized dog models demonstrate that electrical stimulation of the lumbar sympathetic ganglia elicits greater constriction of the hindlimb and splanchnic capacitance vessels than resistance vessels (Hainsworth and Karim, 1976; Karim and Hainsworth, 1976). Because the capacitance vessels function as a blood reservoir, their maximal constriction can dramatically increase preload and cardiac output and are thus a pharmacological target to maintaining blood pressure during anesthesia or septic shock (Hainsworth, 1990; De Backer et al., 2003; Sakka et al., 2007). This speculation is supported by the myocardial adaptations reported herein. Myocardial adaptations to exercise illustrate distinct differences between aerobic and isometric exercise. Aerobic exercise (e.g., swimming, running, etc.) dramatically increases preload and leads to increased left ventricle volumes, mass, and chamber dimensions as the myocardium adapts to volume loading (Morganroth et al., 1975; DeMaria et al., 1978; Maron, 1986). Isometric exercise (e.g., strength training, wrestling, etc.), however, increases afterload *via* vasopressor responses and results in increased wall thickness and mass of the left ventricle without any increase to the internal dimensions or preload – changes to the filling pressure and volume are minimal, but the myocardium thickens in order to generate sufficient force to overcome the increased afterload (Morganroth et al., 1975; DeMaria et al., 1978; Maron, 1986). Skeletal muscle contraction during voluntary lower extremity movement would decrease venous capacitance and increase venous return to the right atrium, thereby increasing preload similar to aerobic exercise. The significant increases to the left ventricle mass, stroke volume, and internal systolic and diastolic dimensions are thus more likely to result from repetitive increases in preload rather than afterload, despite significant increases to the arterial blood pressure observed previously (Aslan et al., 2018; Harkema et al., 2018a,b). And even though there were no significant increases to the end-diastolic volume after scES interventions, there were still significant changes to the diastolic function outcomes that indicate positive changes to the passive diastolic filling pressure (Stoddard et al., 1989).

Given the proximity of the stimulator to the lumbar sympathetic ganglia and that the significant increases we found indicate myocardial adaptation to increased preload, it is possible that scES removes blood from the compliant venous circulation to a greater degree than it elicits arterial vasoconstriction, which led to the significant changes in the cardiac structure, systolic function, and diastolic function after scES intervention. However, there were no significant differences between scES intervention, and in order to understand the effects of CV scES alone and compared with Voluntary and Stand scES, more research is needed. Additionally, investigation into catecholamine release, venous compliance, venous flow and velocity, and arterial diameter and stiffness would elucidate the mechanism by which scES increases preload and afterload.

## Limitations

The data reported in this study were obtained from a heterogenous, young cohort of four individuals with severe cervical spinal cord injury without a statistical control since each participant served as their own control in this pre- and post-measurement study design. The clinically heterogenous injury characteristics make generalization inappropriate, while the small sample makes discrimination between the effects of each scES intervention difficult. However, the significant improvements are promising and justify expanding the research into a larger cohort clinically representative of the SCI population. This would allow us to investigate the effects of scES interventions on cardiac function in lower-level injury, incomplete injury, and in relation to age-related declines in cardiac function.

## Conclusion

We found significant improvements to the ejection fraction and stroke volume, diastolic filling times, and left ventricle dimensions after scES interventions, indicating that scES led to restorative cardiac remodeling in these four individuals with SCI. This has the potential to decrease the secondary health consequences of spinal cord injury and improve quality of life, with implications for improving cardiovascular health and attenuating immobility-related declines in cardiac function. Future studies should investigate this in a larger representative group of SCI participants.

## Data Availability Statement

The raw data supporting the conclusions of this article will be made available by the authors, without undue reservation.

## Ethics Statement

The studies involving human participants were reviewed and approved by University of Louisville Institutional Review Board. The patients/participants provided their written informed consent to participate in this study.

## Author Contributions

BL: writing – original draft, methodology, visualization, and interpretation. SW: methodology, investigation, and writing – review and editing. BU: formal analysis and writing – review and editing. NS and MS: interpretation and writing – review and editing. SH: conceptualization, methodology, writing – review and editing, resources, and funding acquisition. GH: conceptualization, methodology, interpretation and writing – review and editing. All authors contributed to the article and approved the submitted version.

## Conflict of Interest

The authors declare that the research was conducted in the absence of any commercial or financial relationships that could be construed as a potential conflict of interest.
